# Hospital Almoners and Insured Patients

**Published:** 1913-01-18

**Authors:** Edgar S. Kemp


### The Editor's Letter-Box.

### Hospital Almoners and Insured Patients.

*To the Editor of* The Hospital.

Sir,—May I call your attention—if you have not already noticed it—to a statement made by the Treasurer of St. Thomas's Hospital, reported in the *Times* of January 1, as to almoners' work and the Insurance Act?

The hospital, after nearly eight years' experience of an almoner's department, are apparently satisfied that, only through the agency of the almoners, can the question of dealing with insured patients be properly dealt with.—Yours truly,

Edgar S. Kemp.

